# Extremely Low Frequency Electromagnetic Fields impair the Cognitive and Motor Abilities of Honey Bees

**DOI:** 10.1038/s41598-018-26185-y

**Published:** 2018-05-21

**Authors:** S. Shepherd, M. A. P. Lima, E. E. Oliveira, S. M. Sharkh, C. W. Jackson, P. L. Newland

**Affiliations:** 1 Biological Sciences, University of Southampton, Highfield Campus, Southampton, UK; 2 Departamento de Biologia Animal, Universidade Federal de Viçosa, 36570-900 Viçosa, MG Brazil; 3 Departamento de Entomologia, Universidade Federal de Viçosa, 36570-900 Viçosa, MG Brazil; 4 Mechatronics, Mechanical Engineering, University of Southampton, Highfield Campus, Southampton, UK

## Abstract

Extremely low frequency electromagnetic field (ELF EMF) pollution from overhead powerlines is known to cause biological effects across many phyla, but these effects are poorly understood. Honey bees are important pollinators across the globe and due to their foraging flights are exposed to relatively high levels of ELF EMF in proximity to powerlines. Here we ask how acute exposure to 50 Hz ELF EMFs at levels ranging from 20–100 µT, found at ground level below powerline conductors, to 1000–7000 µT, found within 1 m of the conductors, affects honey bee olfactory learning, flight, foraging activity and feeding. ELF EMF exposure was found to reduce learning, alter flight dynamics, reduce the success of foraging flights towards food sources, and feeding. The results suggest that 50 Hz ELF EMFs emitted from powerlines may represent a prominent environmental stressor for honey bees, with the potential to impact on their cognitive and motor abilities, which could in turn reduce their ability to pollinate crops.

## Introduction

A decline of pollinator species can significantly compromise agricultural production^1,2^. Bees contribute approximately 80% of insect pollination, making it crucial to understand and mitigate the causes of current population declines^3^. Pesticide exposure has been identified as a key factor affecting bee health, and ultimately crop pollination abilities^4^. Concerns over increased honey bee colony losses, initially attributed mainly to pesticide use and agricultural intensification, have broadened into studies related to the decline of pollinators in general^3,5^, as well as identifying the varied factors, or stressors that affect pollinator health^6^.

All terrestrial ecosystems are affected by anthropogenic impacts which are expanding inexorably to unprecedented levels^7^. For bees, these impacts include the spread of alien species such as parasitic mites and microsporidia, pesticides, availability of suitable food resource, and rising temperatures and extreme weather events caused by climate change, so that pollinators are increasingly subjected to the simultaneous impacts of multiple stressors^8^. The impacts of different stressors on pollination ecosystem services, particularly when in combination, are currently poorly understood^9^. Improving this situation, and defining solutions, has been identified as one of the most important, and complex, global challenges to reduce pollinator decline^6^.

Extremely low frequency electromagnetic fields (ELF EMFs) are ubiquitous in the environment and are a well-known, but poorly understood stressor. With anthropogenic change, exposure of living organisms to ELF EMFs is increasing and has led to considerable debate as to their adverse health effects^10,11^. Previous studies have shown that chronic exposure to ELF EMFs act as a major stress factor in mammals^12^ that can lead to memory deficits^13^, stress, anxiety behaviour^14^ and depression-like behaviour^15^. Surprisingly few studies have asked whether fluctuating fields at higher intensities, such as ELF EMFs emitted from powerlines, can affect insects. In particular, few studies have addressed the potential impact of acute exposure to ELF EMFs. At ground level ELF EMFs generated under 400 kV transmission lines can reach magnetic flux densities of 100 µT^10^ but flying insects could be exposed to even higher levels closer to the conductor, for example 0.6 mT at 1 m, and 14 mT at 1 cm^16^. Recent studies on the desert locust highlight the potential impact of ELF EMFs on insects, where high levels lead to physiological and behavioural changes as well as increased levels of stress proteins^17^.

Power transmission systems around the world operate commonly at 400 kV. While EMFs are high near 400 kV transmission lines they also remain substantial around lower voltage cables to residential and small commercial properties. Thus, the area around power lines where pollinators may be affected by acute EMF exposure may, therefore, be high. Given what we already know of the effects of EMFs on insects^17^ the opportunity is within our grasp to determine the impact of acute ELF EMFs on important pollinators. Here we focus on the commercially important honey bee and ask how ELF EMFs, at strengths found in the environment, affect their motor, cognitive and feeding abilities. We found that transient exposure to EMF reduces a bee’s ability to learn, reduces their memory retention, affects flight and foraging behavior all of which could potentially reduce their ability to pollinate.

## Results

### Electromagnetic Fields

**Figure 1:**
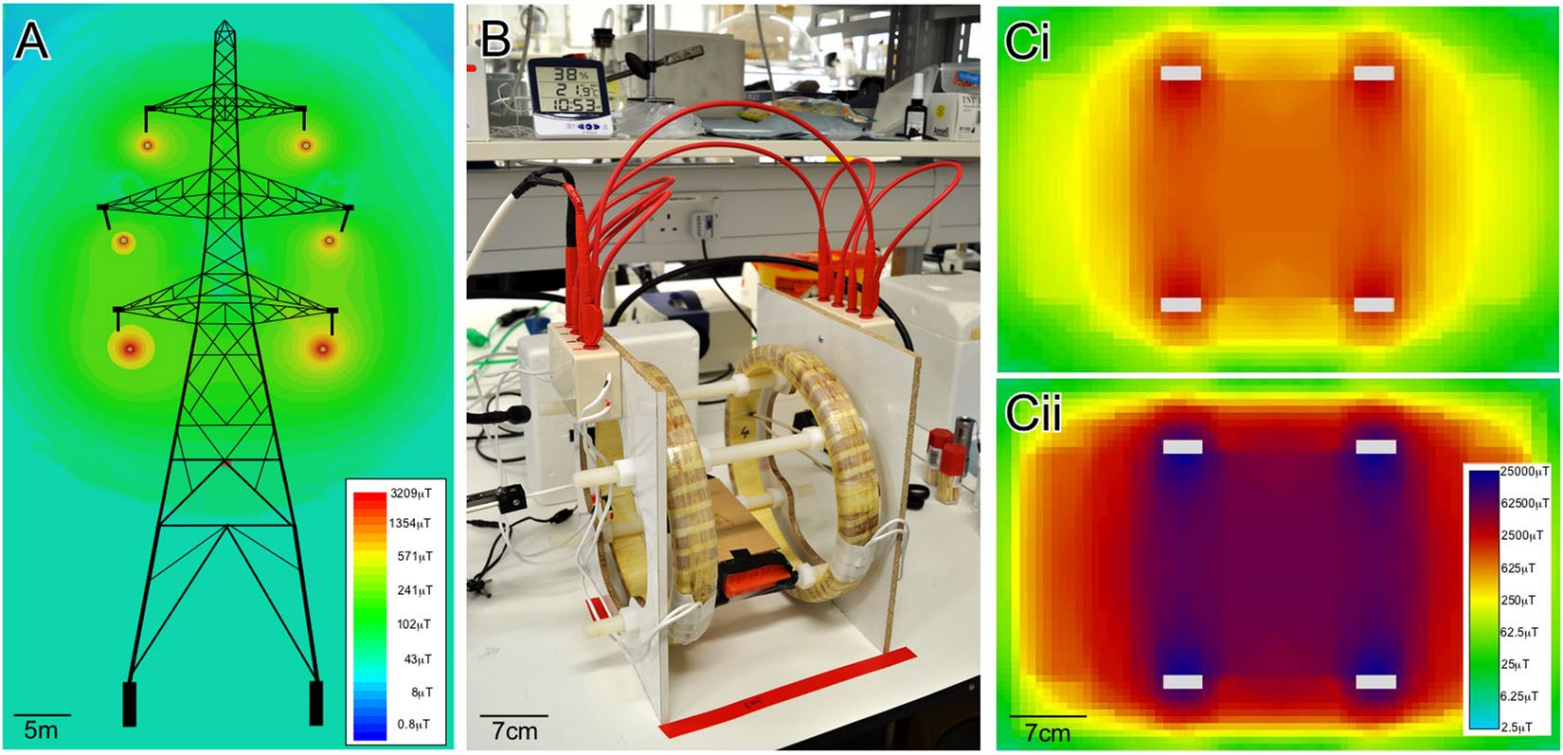
Electromagnetic fields (EMF) around transmission lines and experimental coils. (**A**) 50 Hz EMF distribution around a 400 kV Larger L6 pylon modelled using Maxwell SV modelling software. (**B**) Photograph showing the double-wound copper coils used to generate EMF. (**Ci**) The distribution of 50 Hz EMF around the coils with the voltage set to produce a 1000 μT field at the centre, and (**Cii**) a 7000 μT field at the centre, measured using an Alphalab magnetometer.

The typical clearance between the lowest conductor and the ground of a 400 kV Larger L6 pylon varies between 7.5–12 m depending on tower design, the sag in the conductor between pylons, and the temperature of the conductor, which in turn is dependent on weather and current loading. The ELF EMF is principally dependent on the current loading of the conductors, which can be as high as 3400 A per circuit, and on the Larger L6 pylon there are two parallel circuits each with three phases. Using Ansys Electronic Desktop, MAXWELL, software we modelled the magnitude of the ELF EMFs immediately around Larger L6 Pylon conductors using the highest current rating of 3400 A and found the field at 1 cm to be 3 mT, at 10 cm to be 2.7 mT and at 1 m below the lowest conductor to be 1.2 mT (Fig. 1A). For a 400 kV T-Pylon the fields range from 13.83 mT at 1 cm, to 6.05 mT at 10 cm and 0.6 mT at 1 m. For reference, the average magnetic field 1 m above ground under a Pylon is 5–10 μT, and the maximum is 100 µT^18^. A set of double-wound coils were constructed (Fig. 1B) and the voltage applied adjusted to generate fields at the centre of the coils of 0, 20, 100, 1000 and 7000 μT (Fig. 1Ci,ii) to replicate in the laboratory field strengths present in the environment.

### Learning and Memory

We analysed the effects of acute exposure to ELF EMF on the proboscis extension response (PER) of bees. Bees were exposed EMFs for 1 min immediately following five 1 min conditioning trials. This simulated in the laboratory a realistic scenario of exposure of flying bees in the field crossing an EMF boundary of a powerline immediately after location/returning to a food source.

**Figure 2:**
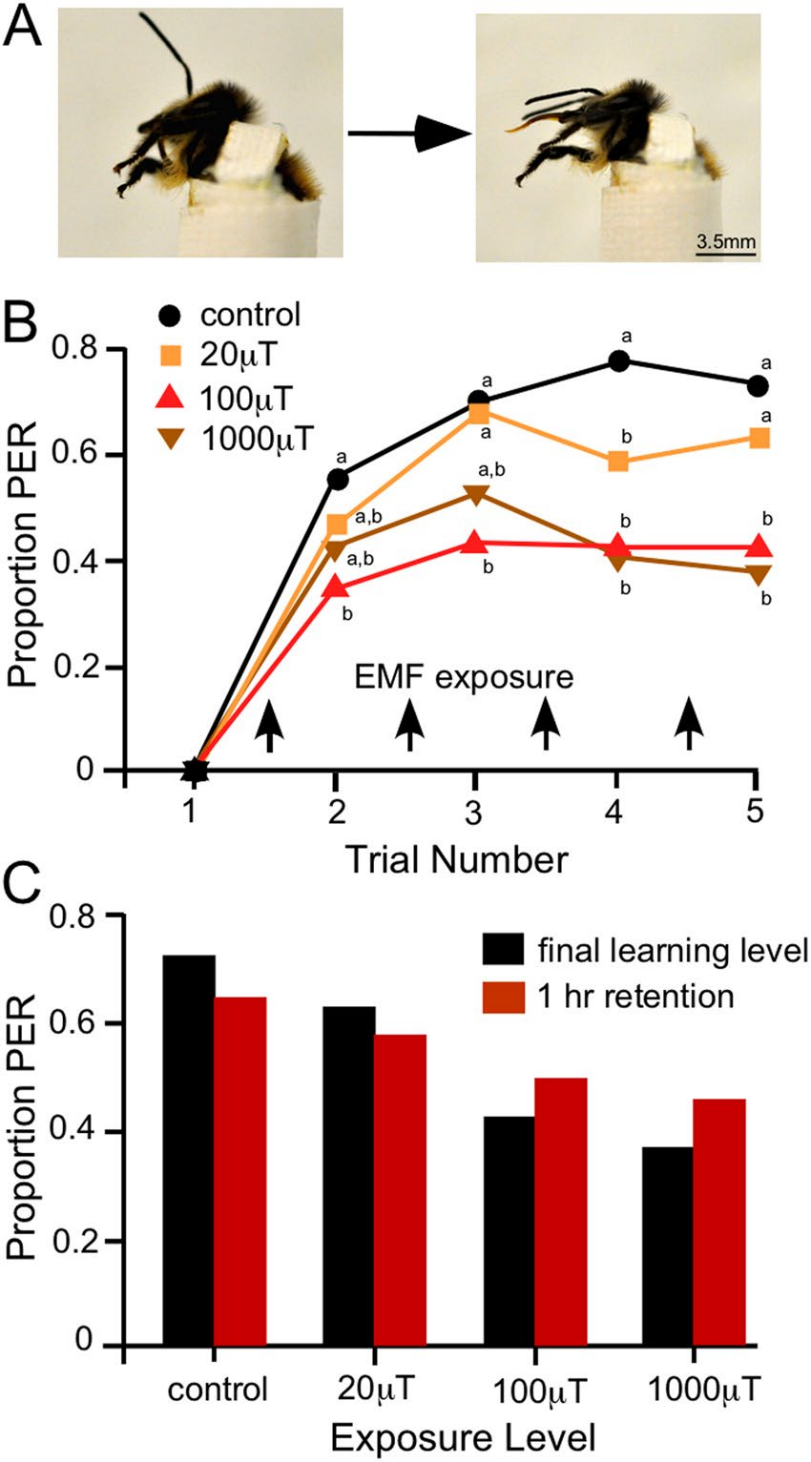
Effect of EMF on the proboscis extension response. (**A**) Photographs showing bee before and during PER. (**B**) PER acquisition of bees over 5 conditioning trials with exposure to differing levels of EMFs or control treatment. The proportion of bees exhibiting PER (response to the CS before US reward) generally increased with trial number. Within each trial, there are no significant differences between treatments with the same assigned letter. (**C**) Levels of PER for final-learning and retention analysis (change between these two levels is compared for memory analysis). The exact proportion of PER responses in the final conditioning trial (trial 5) and the retention trial (1 hr after conditioning) are plotted for each EMF/control treatment. For both (**B**) and (**C**) n = 438 bees (Control, 114; 10 µT, 111; 100 µT, 106; 1000 µT, 107).

The proportion of bees exhibiting PER increased with conditioning trial number. Control bees, which were not exposed to EMFs, showed the highest levels of PER, compared to bees exposed to ELF EMFs of 20, 100 and 1000 µT (Fig. 2B). Control bees reached a peak of 77% PER after trial 4 and attained a final level of 73% PER during trial 5. Bees exposed to 20 µT EMFs showed similar levels of learning acquisition to control bees for trials 2–3, reaching 68% PER in trial 3. During trials 4 and 5, however, lower levels of PER, of 59% and 63%, were expressed compared to control. Bees exposed to 100 µT and 1000 µT ELF EMFs after each conditioning trial exhibited lower levels of PER than control or 20 µT exposed bees for all trials (Fig. 2B). For example, bees exposed to 100 µT reached a peak learning level of 43% in trial 3 and a final learning level of 42%, while bees exposed to 1000 µT EMFs had a peak learning level of 51% in trial 3, and the lowest final learning level of all treatments at 36%.

A GLMM was used to compare the effects of EMF exposure on learning acquisition. EMF exposure for 1 min following each conditioning trial significantly reduced learning acquisition across trials 2–5 (GLMM, *F* = 27.97, d.f. = 3,1688, P < 0.001) with control bees acquiring significantly higher levels of learning than all EMF exposures across the four trials (54–77% acquisition, Bonferroni: P < 0.001) (Fig. 2B). Bees exposed to a 20 µT EMF attained lower learning than controls but significantly higher learning acquisition than 100 µT and 1000 µT exposed bees (47–68% acquisition, Bonferroni: P < 0.001) (Fig. 2B). Learning acquisition did not differ significantly between bees exposed to 100 µT and 1000 µT (100 µT: 35–43%; 1000 µT: 36–51% acquisition, Bonferroni: P = 0.29). These results showed that short-term exposure to EMF impacts on the cognitive abilities of bees by reducing olfactory learning acquisition, and that the magnitude of the effect was dependent on the strength of the EMF.

Some hives performed better in the PER assay than others, as hive of origin also significantly affected learning acquisition (GLMM *F* = 14.03, d.f. = 3,1688, P < 0.001). There was also a significant interaction between ELF EMF exposure and hive of origin (GLMM *F* = 3.68, d.f. = 9,1688, P < 0.001) with all hives having reduced learning acquisition following EMF exposure (Bonferroni: P < 0.001) apart from hive 2. The reduced learning acquisition following EMF exposure of bees from hive 2, however, approached significance (Bonferroni: P = 0.052). Trial number significantly affected learning acquisition (GLMM, *F* = 6.19, d.f. = 3,1688, P < 0.001) as trial 2 had significantly lower rates of acquisition than trials 3–5 (Bonferroni: P = 0.01–0.001) showing that bee learning acquisition usually plateaued by trial 3 in the PER assay (Fig. 2B). There was no significant three-way interaction between EMF exposure, hive of origin and trial number on learning acquisition (GLMM, *F* = 0.56, d.f. = 27,1688, P = 0.97). Similarly, there was no significant two-way interaction between hive of origin and trial number (GLMM, *F* = 0.68, d.f. = 9,1688, P = 0.72) or EMF exposure and trial number (GLMM, *F* = 1.76, d.f. = 9,1688, P = 0.07) on learning acquisition.

EMF exposure significantly reduced the final learning level (after 5 trials) of bees (GLM: χ^2^ = 34.02, d.f. = 3, P < 0.001) compared to controls. For bees exposed to 20 µT EMF 63% exhibited PER in the final learning trial (Fig. 2C) which was significantly lower that the 73% acquisition in control bees (Bonferroni: P = 0.05). The proportion of learned responses in the final trial significantly reduced to 42% following exposure to 100 µT EMFs (Bonferroni: P < 0.001), and 36% after exposure to 1000 µT EMFs (Bonferroni: P < 0.001). 1 hr after the final learning trial a single response test to the CS was carried out to assess how acute EMF exposure affected memory retention (Fig. 2C). During this trial bees still exhibited significantly lower responses to the CS than control bees (GLM: χ^2^ = 9.80, d.f. = 3, P = 0.02). For all EMF exposures however, the proportion of CS responses 1 hr after learning was not significantly different to the proportion of CS response in the final learning trial (pairwise repeated sample McNemar test, P = 0.32–1.00), indicating that memory retention over the 1 hr time period was not affected by EMF exposure during conditioning.

Bees from different hives again showed significant variation in the proportion of PER in the final learning trial (GLM: χ^2^ = 13.42, d.f. = 3, P = 0.004) and in the 1 hr retention trial (GLM: χ^2^ = 27.829, d.f. = 3, P < 0.001), although there was no significant interaction between hive of origin and EMF exposure on the final level of learning (GLM: χ^2^ = 16.88, d.f. = 9, P = 0.06) or for the 1 hr retention trial (GLM: χ^2^ = 10.319, d.f. = 9, P = 0.33).

### Effects of EMFs on Tethered Flight

**Figure 3:**
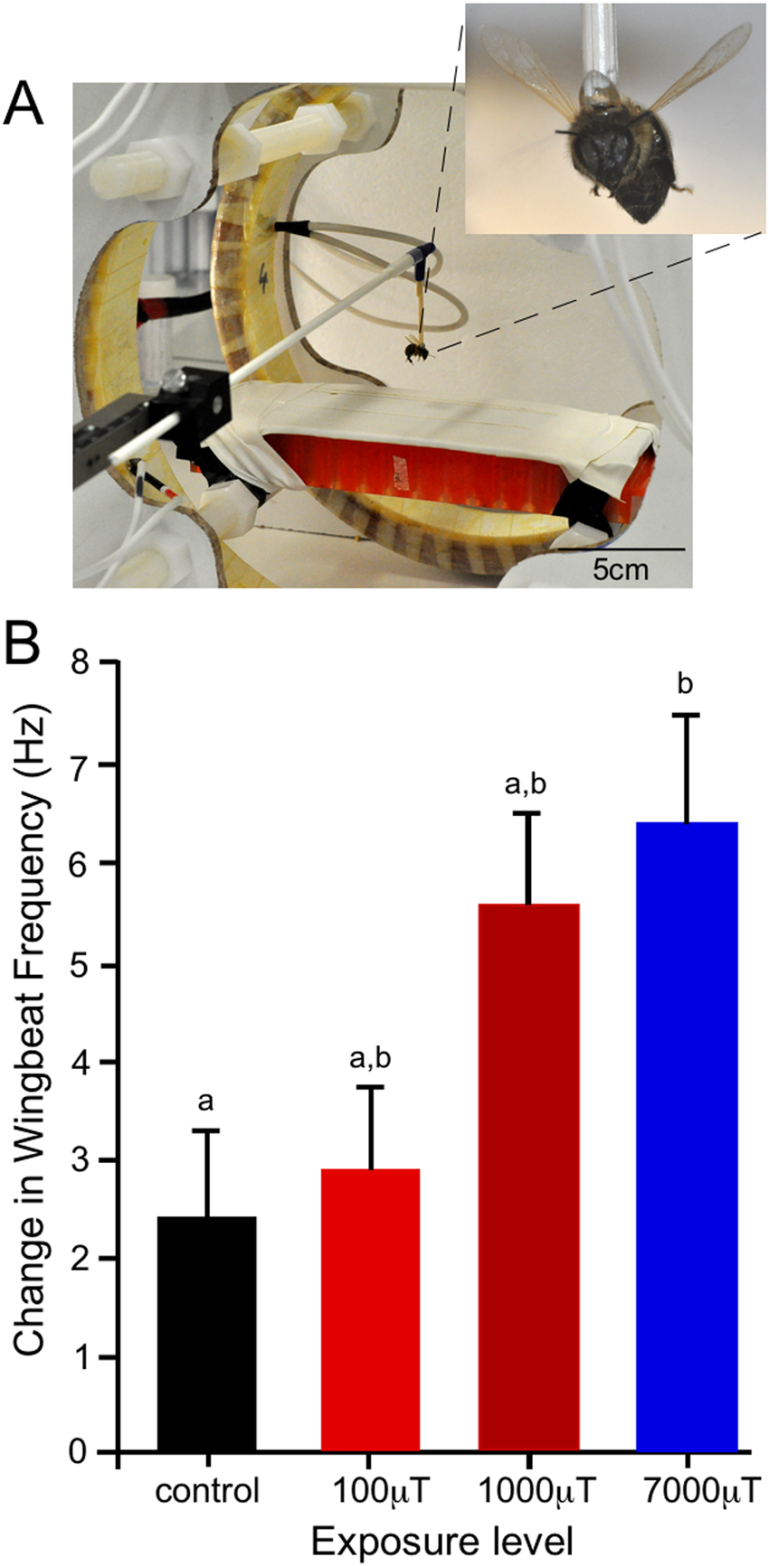
Effect of acute exposure to EMF on tethered flight. (**A**) Image of bee within the coils. (**B**) Changes in wingbeat frequency from control to treatment levels 2.5 s after exposure began for all EMF/control treatments. n = 120 bees (Control: 30, 10 µT: 30, 100 µT: 30, 1000 µT: 30). Mean ± SEM are plotted. There are no significant difference between treatments with the same assigned letter.

To determine the effects of acute ELF EMF exposure on flight, bees were exposed to EMFs during steady tethered flight (Fig. 3A) and changes in wingbeat frequency analysed. For all treatments wingbeat frequency increased within 3 s of exposure onset relative to pre-treatment levels, however, all EMF exposures caused a greater increase in wingbeat frequency than found in control bees (Fig. 3B). Exposure to EMFs during flight significantly increased the wingbeat frequency of bees (ANOVA, F_3,108_ = 4.42, P = 0.006). The increase in wingbeat frequency caused by EMFs was greater with higher intensity exposure levels (100 µT caused an increase of 2.9 ± 0.8 Hz, 1000 µT caused an increase of 5.6 ± 1.0 Hz, and 7000 µT caused an increase of 6.4 ± 1.1 Hz). There were no interactions between hive of origin and treatment (ANOVA, F_6,108_ = 1.10, P = 0.37) nor an effect of hive of origin on a change in wingbeat frequency (ANOVA, F_2,108_ = 1.63, P = 0.20).

### Effects of EMFs on Foraging

**Figure 4:**
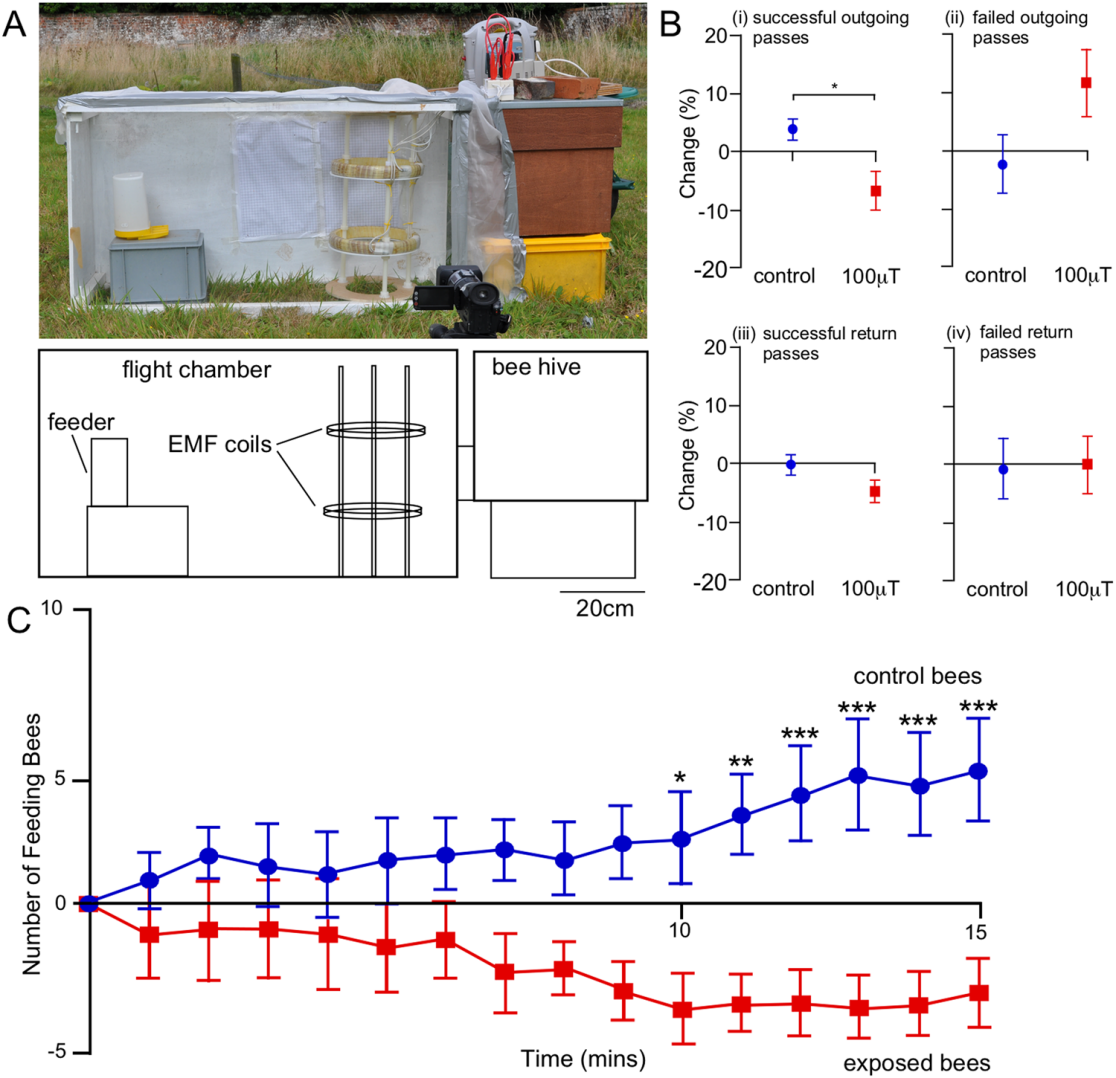
Effect of EMF on foraging flight. (**A**) The experimental arena. (**B**) Change in flight outcomes (successful passes or failed passes) between pre-treatment and treatment levels for control and 100 µT EMF exposure. Exposure to EMF significantly reduced successful outgoing passes (Bi), and significantly increased failed outgoing passes (Bii). EMF exposure had no effect on successful (Biii) and failed return passes (Biv). n = 6 (nucleus hives used as replicates). Mean ± SEM are plotted. (**C**) Number of bees feeding each minute relative to baseline levels (the amount of bees immediately before treatment began) for 100 µT EMF and control treatments. Mean ± SEM are plotted. Asterisks indicate significant differences between treatments at each time point; *p < 0.05, **p < 0.01. ***P < 0.001.

The effects of EMF were analysed on freely moving bees in a flight tunnel in the field. Experimental flight arenas were attached to the front of hives so that bees could fly freely within the arena, but had to fly through a set of coils generating EMF to get to and from the feeder (Fig. 4A). Bees were trained to the feeder within the flight arena, after which pre-treatment levels of flight and feeding data were recorded. Control or EMF treatments were then applied, and the effects of EMFs on foraging assessed.

In free flying bees, exposure to 100 µT EMFs reduced significantly the percentage of successful outgoing flying passes from the hive to the feeder by −6.6 ± 3.2% compared to an increase in successful outgoing passes of 3.8 ± 1.8% under control conditions (t-test, d.f. = 5, P = 0.026) (Fig. 4Bi). Exposure to 100 µT EMF led to an increase in failed outgoing passes by 11.6 ± 5.6% in comparison to a reduction of −2.5 ± 5.1% in control conditions (Fig. 4Bii), which approached significance (t-test, d.f. = 5, P = 0.076). By contrast exposure to 100 µT EMF had no significant effects on the returning passes from the feeder to the hive, for both the percentage of successful passes (control −0.5 ± 1.5%; 100 µT −4.9 ± 1.9%) (Fig. 4Biii) across the treatment area (t-test, d.f. = 5, P = 0.14) and the percentage of failed passes (control −0.8 ± 5.1%; 100 µT −0.1 ± 5.0%) (Fig. 4Biv) across the treatment area (t-test, d.f. = 5, P = 0.90).

Exposure to 100 µT EMFs significantly reduced the number of bees feeding relative to pre-treatment levels compared to control conditions (RM-ANOVA, F_1,5_ = 6.8, P = 0.047) (Fig. 4C). There was a significant interaction (RM-ANOVA, F_14,70_ = 2.79, P = 0.0024) of EMF exposure and time on the number of bees feeding relative to control (Fig. 4C). Overall, time did not affect the number of bees feeding relative to pre-treatment levels (RM-ANOVA, F_14,70_ = 0.29, P = 0.99). As the time after the onset of treatment increased the number of bees feeding increased under control conditions (as more bees learn the location of the food source), but decreased with exposure to 100 µT EMF (Fig. 4C). In control experiments after 15 mins feeding activity increased by 4.5 ± 1.7 bees, which was significantly greater than feeding activity after 15 min of exposure to 100 µT EMF (Bonferroni: P = 0.0001) which decreased by −3.0 ± 1.2 bees.

## Discussion

The results of our analysis show that acute exposure to ELF EMFs impacts upon the motor and cognitive abilities of bees and reduces feeding. We show, for the first time, that acute exposure to ELF EMFs causes a dose dependent reduction in olfactory learning. High levels of ELF EMFs, that can be experienced close to power lines, modify tethered flight by increasing wingbeat frequency. In addition, we show that exposure to low-level fields, at intensities found at ground levels below power lines, significantly reduces the number of successful foraging flights to a food source, and also leads to reduced feeding in bees.

A reduced learning performance can be detrimental to bees^19^. In their natural environment bees must learn and process the location of food, resource quality and type (e.g. colour, smell, shape of flowers), geographic landmarks, as well as the distance and direction of food sources from the hive to communicate to the colony^20^. Differing abilities to learn through olfactory conditioning are highly correlated with natural foraging success in bees^19^, and hence a reduction in learning performance can lead to stress on both the individual and colony level. EMFs reduced learning performance at all levels bees were exposed to, from those of 1000 µT that might be experienced <1 m from a transmission line to 20–100 µT EMFs that will be experienced at ground level. Bees, without any doubt, will be exposed to similar levels of EMFs in the field which are therefore likely to lead to similar cognitive effects where transmission lines are located between hives and food sources.

Greenberg *et al*.^21^, found that honey bee hives exposed to high voltage transmission lines showed increased motor activity, abnormal propolisation, reduced weight gain of hives, queen loss, impaired production of queen cells, decreased sealed brood and poor winter survival. These are all effects that could be caused by poor acquisition of food sources and metabolic inefficiencies. Cognitive behaviour and flight are therefore not the only behaviours ELF EMF pollution may affect and that could lead to colony level stress.

Bees have evolved a magnetosensitive sense to detect the earths static geomagnetic field, and can use that information to navigate. Magnetoreception is well documented in bees, with two potential mechanisms: cryptochrome^22^ and magnetite^23–26^. Clearly a magnetite-based system that has evolved to detect static fields cannot provide directional information within an alternating ELF field, however, it is possible that oscillatory movements of magnetite particles could lead to small oscillatory movements of the abdomen that could be detected and used to generate or modify other behavioural responses, such as those described in this study. Kirschvink *et al*.^27^ suggested, however, that a level of 100 µT is required to discriminate ELF fields at 60 Hz, which is at least an order of magnitude higher than that for static fields^28^, and also significantly higher than the 20 µT level which we found to reduce olfactory learning. Moreover, based on an analysis of the waggle dance of bees, Walker and Bitterman^28^ suggested that the magnetoreceptor system fails to function in EMFs greater than 500 µT, well below the 1000 µT level found near powerlines which we also find significantly affects learning and flight. Together these results suggest that activation of the magnetosensitive alone system may not be the only pathway/mechanism underlying the responses found to acute ELF EMF exposure in this study. It is clear that there is still much to learn regarding the detection of both static and ELF EMFs and that further studies are needed to bridge this fundamental gap in our understanding.

Together our results show that acute exposure to ELF EMFs, at intensities ranging from those at ground level to those near transmission lines, can have profound effects on honey bees. Given that low levels of EMF exposure reduced the numbers of bees feeding, it is possible that EMFs could affect the acquisition of important resources for the colony. In future studies this could be determined by assessing long-term environmental ELF EMF exposure and its effects on resource acquisition, foraging efficacy and resultant colony performance. Reductions in learning, flight and feeding performance, as well as changes in flight activity, can be detrimental not just to the individual bee, but also the colony, as these effects may reduce resource allocation, increase the energy expenditure of honey bees and diminish overall robustness of colonies. We found that there was a increase in wingbeat frequency in bees exposed to EMF, and while relatively small could have an impact on bee health. Insect flight is an energy-demanding motor behaviour^29^ and as a result honey bee lifespan is highly correlated with total flight performance and activity/energy turnover, as shown by the dramatic seasonal relationship between increased honey bee mortality and increased energy expenditure during periods of increased foraging^30^. Any interruption in normal flight behaviour could therefore lead to a greater energy demand for bees exposed to EMF. Moreover, the effects cause by EMF on flight could serve as an aversive stimulus that bees could avoid. There are therefore many questions remaining to be addressed to fully understand how EMFs could impact on bees.

Our results go some way to providing a basis for the observations that ELF EMFs have negative effects on colony stress in bee hives kept under transmission lines^21^, and that bees hived under high voltage powerlines will readily abscond^31^. Other studies have also reported poor success of bee hives kept under powerlines^32,33^ that led to a precautionary federal recommendation in the USA to not keep bee hives under powerlines^34^. The effects of ELF EMFs on insect distributions under and around powerlines may therefore be complex. This is clearly important as powerline strips have been suggested to be important refuges for insect pollinators as they contain large areas of land with beneficial host plants, leading to relatively good pollinator species richness including rare species of bees^35–37^ as well as other important pollinators such as butterflies^38^.

The area around power lines where flying pollinators could potentially be affected by EMFs is relatively high. Whilst foraging ranges of different bee species vary, if the general foraging range of bees is conservatively estimated to be up to 1.5 km^39^ this gives rise to a 3 km corridor around a transmission line within foraging range of bees. In the UK there is 22,643 km of high voltage (400 kV and 275 kV) power lines which means that bee colonies in a 67,929 km^2^ area of land are potentially within foraging range of ELF EMFs. This equates to almost 30% of land in the UK where bees may be exposed to EMFs of 50 µT at ground level and much higher levels closer to the conductor. Local distribution companies own over 279,000 km^40^ of lower voltage 132 kV power lines that also generate EMFs ranging up to 1 mT in close proximity to cables, which can also impact on bees. Thus, the area of land where low to high levels of EMF pollution is within foraging distances of bees is vast.

The impact of these high EMFs on flying insects in general, whose biomass is in significant decline^41^, and pollinators in particular, has been overlooked throughout the debate about the effects of low level EMFs on human health. It is clear that we now need to understand the effects of EMFs on pollinator species in general, the mechanisms underlying these cognitive and motor effects, and the ecological implications of EMF pollution in the field, including impacts on ecosystem services that bees and other insects provide.

## Materials and Methods

Electromagnetic field modelling of the 50 Hz ELF EMF levels around a 400 kV Larger L6 pylon was carried out using Ansys Electronic Desktop, MAXWELL software (Ansys, USA). Modelling (Fig. 1A) provided accurate estimates of the magnitude of the ELF EMFs that were then replicated in the laboratory (20–7000 µT) to determine their effects on honey bees. Field strengths in a similar range to those in the field were then used for laboratory-based experiments.

### Electromagnet Coils

EMFs were generated using custom-made 25 cm (inner diameter) paired wire coils (Fig. 1B). Coils were fixed 14 cm apart for laboratory experiments and 20 cm apart for field experiments. Coils were powered with 240 V AC through Variacs (RS Pro, RS Components, UK) to generate homogeneous 50 Hz sinusoidal EMFs with a range of magnetic flux densities from 10 µT-10000 µT. Magnetic flux densities were measured with a Magnetometer (Model GM2, Alphalab Inc., USA) (Fig. 1C). For control experiments no current was passed through the coils.

### Learning and Memory

Honey bees, *Apis mellifera*, were kept on a campus apiary. In 2015 and 2016, returning forager bees from 4 hives were collected. In the laboratory they were immobilized on ice and secured using adhesive tape (tesa SE, Germany) in 1 ml pipette tips^42^. Bees were fed with a 40% w/v sucrose solution to satiation and maintained overnight at 29 ± 1 °C in a plastic perforated container with wet tissue paper to prevent desiccation.

To analyse proboscis extension reflex responses (PER) an experimental arena (W × D × H = 60 × 45 × 55 cm) was used with a flow through multi-channel odour delivery system. This allowed a constant clean airflow to be supplied to the arena which could be switched to/from the conditioned stimulus (CS) airflow. 8 µl of 97% linalool (Sigma-Aldrich, UK) was pipetted onto filter paper in the CS airflow channel.

PER assays were conducted the morning after collection. Bees were tested for gustatory responsiveness with sucrose solution and those that failed were excluded along with overnight mortalities. For conditioning trials bees were placed individually in the arena and allowed to adapt to the arena for 15 s before being presented with the CS for 10 s. 5 s after CS onset the antennae of a bee was stimulated with 40% sucrose (unconditioned stimulus, US). The bee was allowed to feed on the US for 10 s (pairing CS and US for 5 s), given 30 s clear airflow and then removed from the arena. The inter-trial interval was 10 min for each bee, and each bee was given 5 conditioning trials, followed by a single PER trial 1 hr after the final conditioning trial to assess retention.

If a bee extended its proboscis during CS application (Fig. 2A), but before the US, PER was recorded. Bees that responded to CS in the 1st trial were excluded as conditioning cannot be recorded in bees that already respond to the CS. A ‘gustatory response’ was recorded if a bee only extended its proboscis during the US. If a bee did not extend its proboscis ‘no response’ was recorded. Bees that failed gustatory responsiveness were excluded from analysis.

To analyse the effects of ELF EMFs on associative learning, bees were exposed to EMFs for 1 min immediately following each of the 5 × 1 min conditioning trials. This simulated in the laboratory a realistic scenario of exposure of bees in the field crossing an EMF boundary of a powerline immediately after locating/returning to a food source. Bees were exposed to 3 different EMF levels (20 µT, 100 µT, and 1000 µT) or control exposures that represented levels found in the natural environment, those at ground level below a powerline and those 1 m from a cable (Fig. 1A).

In total 549 bees survived overnight from the 4 hives for learning and memory assessment. Of these 74 (13.5%) were excluded from trials for failing gustatory responsiveness and 37 (6.7%) for a pre-learned response to the CS. Therefore 438 bees were included in trials to determine the effects of ELF EMFs on learning and memory.

### Tethered Flight

Following collection as described above, hairs on the dorsal side of the thorax of bees were removed using a scalpel, to provide an attachment point. Bees were then attached to a tether using UV activated glue (Bug-Bond™, Veniard Ltd., Croydon, UK). The tether allowed bees to be placed on, and raised from, a platform in the centre of the electromagnet coils (Fig. 3A). Raising bees vertically from the platform initiated flight. A high-speed video camera (MotionScope 1000 S, Redlake Imaging, CA, USA) was used to record flight at 1000 fps. Following 5 s of consistent flight an EMF was applied and the video camera triggered to capture flight 1 s prior to EMF exposure and 3 s after exposure. Bees (120 in total, n = 30 for each treatment, from 3 hives) were exposed to 1 of 3 different EMF (100 µT, 1000 µT, and 7000 µT) or control exposures. High-speed video was analysed to determine wingbeat frequencies of bees 0.5 s before EMF exposure (pre-treatment) and 2.5 s after the EMF onset (treatment). To determine the impact of acute EMF exposure on bee flight, the change in wingbeat frequency from pre-treatment to treatment was calculated for each bee, for both control and exposed bees.

### Foraging Flight and Feeding

Foraging experiments were conducted with bees from 6 nucleus hives in the field at Southampton Science Park in summer 2016. Flight tunnels (W × L × H = 40 × 140 × 69 cm) were attached to the front of hives so that bees could fly freely in the tunnel. A feeder was placed at the end of the tunnel, and electromagnet coils were placed 20 cm from the hive and 80 cm from the feeder, so that bees had to fly through the coils to get to and from the feeder (Fig. 4A). The feeder was filled with 100 ml of honey, and 700 ml of 50% w/v sucrose solution. Bees were given 30 min to locate the feeder, and for activity in the tunnel to increase. Subsequently, a 15 min recording of baseline feeding and flight levels was made with no EMF exposure. Following this, 15 min recordings of flight and feeding levels during control or 100 µT EMF treatments were made, allowing the change in feeding and flight from baseline levels during each treatment to be determined. Both treatments (control or EMF) were applied to each nucleus hive, with experiments conducted at least 7d apart, and the order in which they were applied alternated for each nucleus hive (hive 1,3,5: control first, EMF 1 wk later; hive 2,4,6: EMF first, control 1 wk later).

The number of bees feeding every minute during pre-treatment and treatment times was recorded. For analysis the number of feeding bees in the final time point before treatment initiation was used as a baseline value, and all changes relative to the baseline value for each minute in the treatment time period recorded, such that changes in feeding activity in the treatment time period for control *versus* EMF treatments could be compared. The effects of exposure to EMFs on feeding were analysed on 3,699 feeding events recorded during pre-treatment and treatment periods for bees from the 6 hives combined.

A digital video camera (HDR-HC1, Sony, Japan) was used to record all bee flights in an area (40 × 50 × 69 cm) around the coils both in the pre-treatment and treatment periods. Videos were subsequently analysed and the outcomes of all flights were recorded. Bee flights that began on one side of the area and exited from the opposite side were defined as *successful passes*. All other flights that did not fulfil this criterion (i.e. turned around in flight/landed), were defined as *failed passes*. The direction of the flight was also recorded. Changes in the percentages of successful/failed passes between pre-treatment and treatment periods for control and EMF exposures were compared to determine the effects of EMFs on flight performance. 2,919 bee flights were recorded from bees from 6 nucleus hives to analyse the effect of EMFs on free foraging flight.

### Statistics

A generalized mixed effect model (GLMM) was used to analyse the effects of EMF treatment on learning acquisition (PER), including ‘hive of origin’ and ‘trial number’ as interactive factors, while generalized linear models (GLM) were used for the analysis of final learning level and 1 hr post conditioning levels, comparing ‘EMF exposure’ and ‘hive of origin’ interactions. For both models binomial error structure and logit link function were used. To determine the effects of EMFs on memory the proportion of PER responses in the final learning trial were compared to the proportion of PER responses in the PER retention trial within each treatment group, using a pairwise repeated samples McNemar test. Analysis of Variance (ANOVA) was used to assess the effects of EMFs on the flight responses, with a two-way structure to include ‘hive of origin’ as an interaction factor, and change in wingbeat frequency as the dependent variable.

For field-based experiments, nucleus hives were used as replicates to avoid pseudoreplication. To determine the effects of EMFs on free flight the percentage change in flight outcomes were compared for control *versus* EMF treatments using a paired-samples t-test. To determine the effects of EMFs on feeding a two-way repeated-measures ANOVA was used, with time and EMF treatment as repeated measures, and the number of bees feeding relative to the pre-treatment period as the dependent variable. Where appropriate pairwise contrasts with Bonferroni adjusted significance were used in *post-hoc* analyses. Where necessary for all statistical analyses the homogeneity of variance and normality assumptions were tested and no data transformations were required. All analyses were carried out using SPSS v. 24 (IBM SPSS Inc., USA).

### Data availability

The data is available from 10.5258/SOTON/D0415.
